# A Novel, Diffusely Infiltrative Xenograft Model of Human Anaplastic Oligodendroglioma with Mutations in *FUBP1*, *CIC*, and *IDH1*

**DOI:** 10.1371/journal.pone.0059773

**Published:** 2013-03-19

**Authors:** Barbara Klink, Hrvoje Miletic, Daniel Stieber, Peter C. Huszthy, Jaime Alberto Campos Valenzuela, Jörg Balss, Jian Wang, Manja Schubert, Per Øystein Sakariassen, Terje Sundstrøm, Anja Torsvik, Mads Aarhus, Rupavathana Mahesparan, Andreas von Deimling, Lars Kaderali, Simone P. Niclou, Evelin Schröck, Rolf Bjerkvig, Janice M. Nigro

**Affiliations:** 1 Institut für Klinische Genetik, Medizinische Fakultät Carl Gustav Carus, Technische Universität Dresden, Dresden, Germany; 2 Department of Biomedicine, University of Bergen, Bergen, Norway; 3 The Gade Institute, Department of Pathology, Haukeland University Hospital, Bergen, Norway; 4 Centre de Recherche Public de la Santé, Luxembourg; 5 Department for Histology and Embryology, School of Medicine, University of Rijeka, Rijeka, Croatia; 6 Institut für Medizinische Informatik und Biometrie, Medizinische Fakultät Carl Gustav Carus, Technische Universität Dresden, Dresden, Germany; 7 Clinical Cooperation Unit Neuropathology, German Cancer Research Center (DKFZ), Heidelberg, Germany; 8 Oncomatrix Research Laboratory, Department of Biomedicine, University of Bergen, Jonas Bergen, Norway; 9 Department of Clinical Medicine, Section Neurology, Haukeland University Hospital, Bergen, Norway; 10 Department of Neurosurgery, Haukeland University Hospital, Bergen, Norway; 11 Department of Surgical Sciences, University of Bergen, Bergen, Norway; 12 Department of Neurosurgery, Oslo University Hospital, Oslo, Norway; 13 Department of Neuropathology, Institute of Pathology, Ruprecht-Karls-University Heidelberg, Heidelberg, Germany; University of Chicago, United States of America

## Abstract

Oligodendroglioma poses a biological conundrum for malignant adult human gliomas: it is a tumor type that is universally incurable for patients, and yet, only a few of the human tumors have been established as cell populations *in vitro* or as intracranial xenografts *in vivo*. Their survival, thus, may emerge only within a specific environmental context. To determine the fate of human oligodendroglioma in an experimental model, we studied the development of an anaplastic tumor after intracranial implantation into enhanced green fluorescent protein (eGFP) positive NOD/SCID mice. Remarkably after nearly nine months, the tumor not only engrafted, but it also retained classic histological and genetic features of human oligodendroglioma, in particular cells with a clear cytoplasm, showing an infiltrative growth pattern, and harboring mutations of *IDH1* (R132H) and of the tumor suppressor genes, *FUBP1* and *CIC*. The xenografts were highly invasive, exhibiting a distinct migration and growth pattern around neurons, especially in the hippocampus, and following white matter tracts of the corpus callosum with tumor cells accumulating around established vasculature. Although tumors exhibited a high growth fraction *in vivo*, neither cells from the original patient tumor nor the xenograft exhibited significant growth *in vitro* over a six-month period. This glioma xenograft is the first to display a pure oligodendroglioma histology and expression of R132H. The unexpected property, that the cells fail to grow *in vitro* even after passage through the mouse, allows us to uniquely investigate the relationship of this oligodendroglioma with the *in vivo* microenvironment.

## Introduction

Until recently, adult human oligodendrogliomas have maintained a degree of anonymity among human cancers, despite hallmark genetic losses of chromosomes 1p and 19q that signaled a unique biological and clinical nature divergent from other gliomas [1]. Tumor suppressor genes targeted by these losses have only recently been identified, *FUBP1* and *CIC*, and are involved in *MYC* expression and repression of receptor tyrosine kinase (RTK) signaling, respectively [2],[3],[4],[5]. However, it was a single point mutation, that of the isocitrate dehydrogenase genes (*IDH1/2*), that suddenly thrust these tumors into the center of more general scientific and clinical interest at multiple levels [6],[7]. Biologically, these mutations corrupt a metabolic pathway and endow the enzymes with a new function that has the overall effect of generating detectable levels of a normally rare metabolite, 2-hydroxyglutarate (2HG) [8]. Elevated levels of 2HG have been shown to influence methylation patterns of histones and DNA and thereby, inhibit differentiation [9],[10]. Clinically, this mutation is associated with a more favorable prognosis, and it is more ubiquitously found early in the genesis of adult lower grade malignant gliomas and secondary glioblastoma (GBM) [7],[11],[12],[13]. The mutations have subsequently become important as diagnostic tools, in addition, because 2HG is visible by mass resonance spectroscopy (MRS) in patients [14],[15],[16], and an antibody against the most common mutation, R132H, has been developed that unequivocally recognizes individual tumor cells [17].

Although oligodendroglioma with its unique mutational constellation generally follows a less aggressive clinical course than GBM, it is still a fatal disease. Only recently, however, have a few tumors of oligodendroglial histology been propagated *in vitro* or as intracranial xenografts *in vivo* [18],[19],[20],[21],[22]. Of the five reported, genetically characterized intracranial xenografts, all had 1p/19q co-deletion, but only one, which was an anaplastic oligoastrocytoma, or a mixed glioma, was found to express a mutated IDH protein, R132H [22]. Therefore, *in vivo* models that accurately reflect tumors of pure oligodendroglioma histology, and in particular, tumors with *IDH* mutation, are still greatly needed to represent this disease, and more broadly, human gliomas with *IDH* mutations.

Here, we report the propagation of a WHO grade III oligodendroglioma as an intracranial mouse xenograft that histologically and genetically replicates features of a classic oligodendroglioma, including expression of the R132H protein. Interestingly, the primary tumor or xenografts did not expand *in vitro*, even though 1p/19q co-deletion with underlying mutations in both *FUBP1* and *CIC* was present, as revealed by aCGH and exome sequencing.

## Materials and Methods

### Ethics statement

Patient material was obtained from surgeries performed at the Haukeland University Hospital (Bergen, Norway). Written consent was obtained from patients with procedures that were approved for the project (project number 013.09) by the Regional Ethics Committee (Bergen, Norway). All animal protocols were approved by authorities in an AAALAC accredited facility at the Haukeland University Hospital and in accordance with the national regulations of Norway (project numbers 2010 2658 and 2011 3079).

### Dissociation

Tumor tissue (<1 g) was gently cut with scalpel blades, rinsed in Hank's buffered saline (HBSS), and enzymatically dissociated at 37°C for one hour, or until single cells could be observed, in 5 ml of HBSS containing Liberase DH (0.25 mg in 5 ml; Roche) and 100 μl DNAse (2% solution in HBSS; Sigma). DMEM with 10% serum was added, and the enzyme mixture was removed after centrifugation. The sample was resuspended in DMEM with 10% serum, gently triturated with a 10 ml plastic pipet, and filtered through a 70 μm filter. Blood cells were subsequently lysed with EasyLyse (Dako), and tumor cells were washed thoroughly two times with 20 ml of Dulbecco's phosphate buffered saline without calcium and magnesium.

### Cell culture

Dissociated tumor cells were resuspended in Neurobasal Medium, B27 supplement, Glutamax (Life Technologies), and FGF2 (20 ng/ml), and EGF and/or PDGFA (20 ng/ml; Peprotech) over non-treated culture flasks (Nunc). Medium referred to as NBM is Neurobasal Medium plus B27 supplement and Glutamax. Medium was changed once weekly. All cell populations used for implantation or culture experiments were fingerprinted to confirm tumor origin. Hippocampal neuronal preparations were prepared from hippocampi isolated from day old rat newborns. Hippocampi were incubated with papain (Worthington) at 37°C for 15 min and resuspended in MEM with 10% serum. A single cell suspension was made by trituration of the tissue with a fire polished glass pipet. Cells were plated in NBM but without growth factors for one week. Tumor spheres were then added with NBM containing FGF2 (20 ng/ml).

### Implantations

Tumor cell aggregates were implanted into 6 to 8 week old eGFP NOD/SCID mice of either gender. Coordinates for implantation were 1.5 mm to the right and 0.5 mm behind the bregma suture. Maximum penetration of the syringe was 2.0 mm into the tissue with injection of the cells at 1.5 mm. First generation refers to xenografts established in mice from the primary patient culture whereas second generation (and any generation thereafter) refers to tumors established from human cells already passaged through mice. All animals were allowed to live until they became symptomatic (lordosis, weight loss, neurological symptoms, or changes in the appearance of fur). For each animal, either a section or the whole brain was formalin fixed and examined on H&E by a neuropathologist to identify cases where oligodendroglioma had developed. NOD/SCID mice are prone to infection and tumor pathologies, or simply old age, which are all possible causes of symptoms or death in the absence of apparent engraftment [23].

### Magnetic Resonance Imaging (MRI)

MRI was performed with a 7T small animal scanner with a circular mouse head transmit/receive coil (PharmaScan; Bruker BioSpin MRI, Ettlingen, Germany). A T2 weighted (T2w) RARE sequence was acquired with Paravision software using the following parameters: TR 3500 ms, TE 36 ms, 4 averages, FOV 2.0×2.0 cm, matrix size: 256×256, slice thickness: 1 mm, and scan time: 5 minutes 36 seconds.

### Immunohistochemistry

After de-paraffinization, formalin fixed sections were pretreated by heat denaturation for 15 min in 10 mM Citrate buffer, pH 6.0. Primary antibodies were incubated with sections overnight at 4°C, and detection was performed using a biotinylated secondary antibody and amplification of the signal with Vectastain ABC Reagent (Vector Labs). Antibodies used were the following: R132H IDH1 (1∶30; Dianova, DIA-H09), Ki67 (1∶100; Dako, M7240), human nestin (1∶100; Millipore, MAB5326), human GFAP (1∶100; Abcam, AB7806), mouse nestin (1∶100; Millipore, MAB353), mouse GFAP (1∶100; Dako, Z0334), PDGFRA and phospho-PDGFRA (1∶100; Cell Signaling, 3164 and 2992), Iba1 (1∶500; Wako, 019–19741), CXCL12 (1∶100; R&D Systems, MAB350) and STEM121 (1∶500; StemCells).

### DNA isolation

DNAs were isolated from patient material or xenografts that had been immediately frozen in liquid nitrogen subsequent to surgery. Frozen sections were prepared, and every fifteenth section was stained with H&E to ensure >60% tumor cell content. DNA was obtained from frozen patient blood (−20°C) by isolating nuclei with Buffer C1 from the Genomic DNA Buffers Kit (Qiagen). Samples were treated with proteinase K overnight at 50°C in ATL buffer (Qiagen) and then DNAse free RNAseA (Fermentas) for 5 min at room temperature. Reactions were extracted with phenol:chloroform:isoamyl alcohol 25∶24∶1 saturated with 10 mM Tris, pH 8.0, 1 mM EDTA (Sigma) and precipitated in 2.5M NH_4_OAc and 2.5 volumes of 100% ethanol. DNAs were resuspended in Nuclease-Free water (Qiagen).

### CGH+SNP arrays and data analysis

DNA was digested using the restriction enzymes RSA1 and Alu1 and labeled using the BioPrime aCGH Genomic Labeling Kit (Invitrogen) and Cy3 and Cy5 dyes (GE Healthcare), following standard protocols for Agilent CGH+SNP. A female HapMap sample with a known genotype (European female, NA12878_V1) provided by the Coriell Repository (Camden, NJ, USA) was used as a reference for each of the CGH+SNP experiments. Labeled DNA was competitively hybridized to SurePrint G3 Human 2×400 k CGH+SNP microarrays (G4842A, Agilent Technologies) following standard Agilent protocols. The slides were scanned at 3 μm resolution using the Agilent High-Resolution Microarray scanner, and the image data were extracted using Feature Extraction (Agilent Technologies). Feature extraction files were imported into Genomic Workbench 7.0 (Agilent Technologies) for visualization and analysis. For CGH, after diploid centralization and GC correction, aberrations were called using the ADM2 algorithm with a threshold setting of 20, centralization on with threshold of 25 and an aberration filter min Probes = 5 and minAvgAbsLogRatio = 0.35 for amplifications and deletions.

### Exome Sequencing

Agilent SureSelect target enrichment kit (SureSelect Human All Exon 50 Mb kit, Agilent) was used for exome library construction according to the manufacturer's protocol. Captured DNA libraries were sequenced on the Illumina HiSeq2000 platform. Reads were aligned to the human reference genome (hg19) with BWA. Duplicate tags were removed. Point mutations, and insertions and deletions (INDELs) were identified using Samtools (version 0.1.18) with default parameters. Somatic variations were then validated in IGV browser 2.1.22.

### PCR and Sequencing

Standard methods for PCR and Sanger sequencing were performed according to the manufacturer's procedures (Applied Biosystems). Primers for PCR were as follows: IDH1 forward,

5′-AATGAGCTCTATATGCCATCACTG-3′; IDH1 reverse, 5′-TTCATACCTTGCTTAATGGGTGT-3′ [24]; IDH2 forward, 5′-CAGAGACAAGAGGATGGCTAGG-3′, IDH2 reverse, 5′-GTCTGGCTGTGTTGTTGCTTG-3′ [7], CIC forward, 5′-TAGAATGCAGTGAGGGCTTG-3′; CIC reverse, 5′-TTGGAGGGAAAGATGTCTGC-3′; FUBP1 forward, 5′-TGCTGACTAGTAATGATACATTTTCC-3′; FUBP1 reverse, 5′-GGCCCATTTAATTGTGACCA-3′. For IDH1, a separate sequencing primer was used, 5′-GCCATCACTGCAGTTGTAGGTTA-3′ [24].

### DNA fingerprinting

The AmpFlSTR Profiler Plus PCR Amplification Kit (Applied Biosystems) was used according to the manufacturer's protocol. This kit amplifies nine tetranucleotide short tandem repeat (STR) loci and the amelogenin (gender determination) locus in a single reaction. The samples were run and allele sizes interpreted on an ABI3100 Genetic Analyzer (Applied Biosystems). The fingerprinting profiles were run through the online international reference STR profile database for human cell lines at the German collection of microorganisms and cell cultures DSMZ (www.dsmz.de). Profiles were analyzed to determine that they matched the original biopsy, and that they did not match any of the cell lines listed in the DSMZ STR database.

### 2-hydroxyglutarate assay

2HG concentrations were measured with an enzymatic assay based on the finding that *IDH* mutations generate the D-2-hydroxyglutarate (D2HG) form of 2HG [25],[8]. This assay exploits the activity of the enzyme D-2-hydroxyglutarate dehydrogenase from Acidaminococcus fermentans which catalyzes the conversion of D2HG to α-ketoglutarate (αKG) in the presence of nicotinamide adenine dinucleotide (NAD+). NAD+ becomes reduced to NADH during this reaction. Determination of D2HG concentration is based on the detection of stoichiometrically generated NADH from this reaction. A second enzyme, diaphorase, is used to convert the non-fluorescent substrate, resazurin, to a fluorescent product, resorufin, under consumption of NADH. Fluorometric detection was carried out on a plate reader with excitation at 540+/−10 nm and emission of 610+/−10 nm.

## Results

### Engraftment of a primary oligodendroglioma biopsy in eGFP NOD/SCID mice

**Figure 1:**
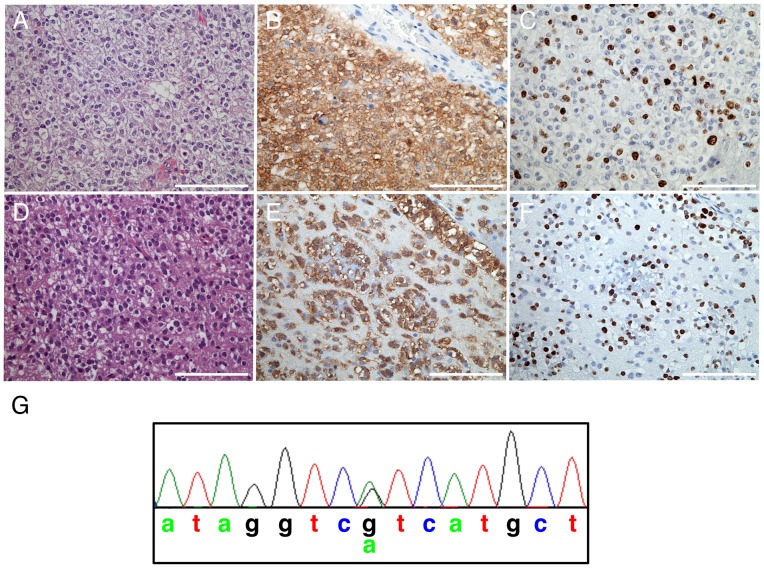
Classic histogological and genetic features of oligodendroglioma in first generation xenografts. (A) H&E section of the primary human tumor; (B) R132H immunostaining; (C) Ki67. (D) H&E section of a xenograft; (E) R132H and (F) Ki67 on successive slides. (G) Sequence of codon 132 demonstrating heterozygosity in the xenograft. Scale bars 100 μm.

A WHO grade III oligodendroglioma was obtained from a surgery performed on a 39 year-old female patient with a history of oligodendroglioma. The case had been originally diagnosed as a grade III oligodendroglioma, when the patient was 35 years old, and FISH analysis performed at that time indicated that the case harbored losses of 1p and 19q, the hallmark genetic changes of human oligodendrogliomas. The patient was treated with radiation and chemotherapy. The present recurrence in 2010 was a highly cellular tumor that retained the original histologic features of the case, including round nuclei with a clear cytoplasm, microvascular proliferation, and a high proliferation index (Figure 1A, B, C).

At the time of the recurrence, approximately one gram of the tumor tissue was enzymatically dissociated, and cell aggregates or spheres were allowed to form in serum free medium containing FGF and EGF in suspension culture. Small cell aggregates formed immediately following dissociation and resuspension in medium. Intracranial implantations were subsequently performed with twenty-five of these spheres containing approximately 1000 cells on two sets of six to eight week old eGFP NOD/SCID mice in order to establish first generation tumors [26]. The first four animals were implanted with cells from the patient tumor that had been in culture for one month, and three additional animals were implanted with remaining spheres from the same culture, but one month later.

First generation tumors arose in animals from both implantations, three out of four mice from the first implantation at one month and one out of three from the second implantation at two months. H&E sections from three of these mice revealed a diffusely infiltrating tumor pathology characteristic of oligodendroglioma (Figure 1D). Symptoms appeared in the first mouse at 267 days, or nearly 9 months after implantation, whereas the next two animals were symptomatic at 312 and 342 days after implantation.

Immunohistochemistry demonstrated that R132H was expressed in the xenograft (Figure 1E) as in the corresponding patient tumor (Figure 1B). In fact, all three cases were positive for R132H demonstrating that R132H was stably expressed in multiple xenografts (data not shown). Immunostaining with the R132H antibody also revealed the presence of a few tumor cells in the corpus callosum of one animal where oligodendroglioma was not histologically evident at 221 days (data not shown). Sanger sequencing of the DNA demonstrated that the R132H mutation was still heterozygous (Figure 1G). To determine the percentage of proliferating human tumor cells, sections were stained with a human specific antibody against Ki67. Nearly 50% of cells were proliferating in the xenograft (Figure 1F) whereas 25% of cells were Ki67 positive in the original patient tumor (Figure 1C).

### Serial passaging of xenograft material

**Figure 2:**
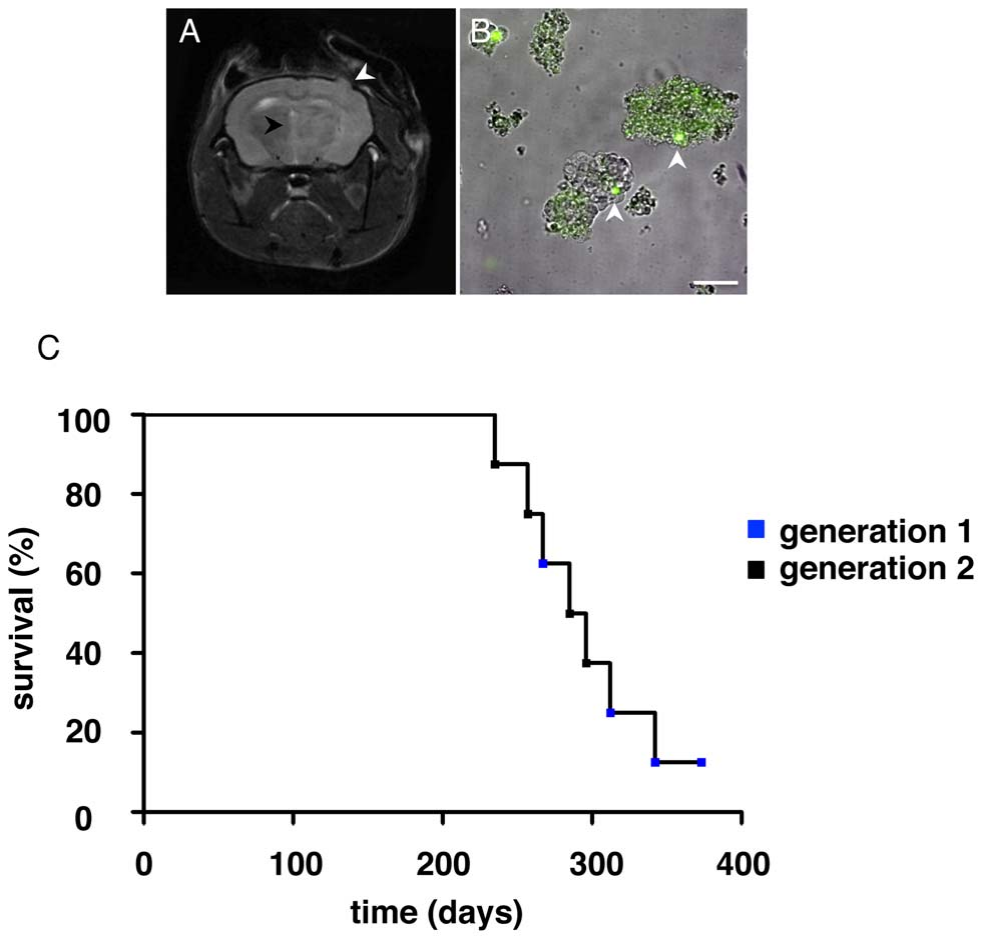
Serial passaging of the xenograft. (A) T2 weighted MRI of affected first generation animal; (B) spheres obtained from dissociated tumor from a eGFP NOD/SCID positive mouse; (C) Kaplan-Meier survival curve of animals with oligodendroglioma when sacrificed in days. All animals were allowed to live until symptomatic or death. Animals without any evidence of oligodendroglioma were not included in the curve. The animal at 374 days in the first generation was included as a censured event because the animal was sacrificed due to the positive MRI rather than being symptomatic. White arrows highlight the implantation site in A and individual eGFP positive cells within spheres in B. Black arrow in A highlights the midline. Blue and black boxes in C represent animals from first and second generations, respectively. Scale bars 100 μm.

MRI performed at 374 days on the fourth, asymptomatic animal revealed that tumor was present based on distortion of the midline and enlargement of the cortex (Figure 2A). Upon sacrifice, the brain was obviously enlarged, and the tumor (implantation site) and contra-lateral hemispheres were dissociated independently. Spheres formed immediately upon re-suspension in serum free medium with FGF and EGF, but only with material obtained from the ipsilateral hemisphere. Distinction of tumor cells was evident based on the fact that this animal was eGFP positive. eGFP fluorescing stromal cells could be seen intermingled in these spheres (Figure 2B).

Spheres from this single mouse were used to create second generation xenografts. Nine new animals were again each implanted with about twenty-five of these spheres containing approximately 1000 cells (Figure 2B). Four out of nine mice developed oligodendroglioma. The time frame for development of xenografts from both generations is represented graphically by a Kaplan-Meier curve (Figure 2C).

### R132H *IDH* mutation and infiltration of tumor cells along perineuronal and perivascular spaces

**Figure 3:**
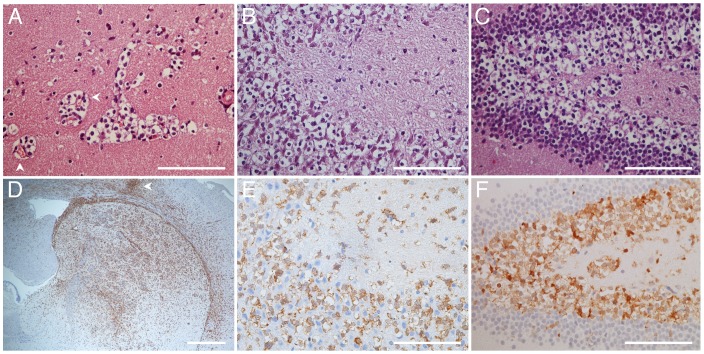
Immunostaining for R132H emphasizes the infiltrative nature of the tumor cells and confirms invasion along neurons in the hippocampus. (A) H&E of oligodendroglioma cells gathered around vasculature; (B) and (C) H&E of xenografts accumulating near neurons in the CA3 region of the hippocampus and the dentate gyrus. (D) Sections from xenografts were immunostained for R132H and taken at low magnification; (E) and (F) R132H immunostaining highlighting tumor cells in the CA3 region of the hippocampus and the dentate gyrus. White arrows in A and D show blood vessels and implantation site, respectively. Scale bars A, B, C, E and F, 100 μm; D 500 μm.

Tumors from both generations exhibited a diffusely infiltrating pathology and had round tumor nuclei and clear cytoplasm that are typical of oligodendroglioma (Figure 1D; Figure 3A, B, C). Endothelial cell proliferation was not evident in either generation; however, tumor cells were observed to cluster around vasculature (Figure 3A). Furthermore, areas of perineuronal infiltration (satellitosis) were evident, a feature that is consistent with the histological features of this tumor type in human tumors (Figure 3B, C).

*IDH* mutation presents a unique opportunity to unequivocally distinguish tumor cells from normal cells in histological sections [17]. We were able to observe that individual cells could migrate far from the implantation site and also along white matter tracts in the corpus callosum and into the contra-lateral hemisphere. The R132H staining illustrates the fundamentally, highly infiltrative nature of this tumor type and thus the therapeutic challenges to treating these patients (Figure 3D). Furthermore, we were able to confirm that the cells accumulating around neurons in the hippocampus were indeed tumor cells (Figure 3E, F).

### Oligodendroglioma cells produce 2HG

**Figure 4:**
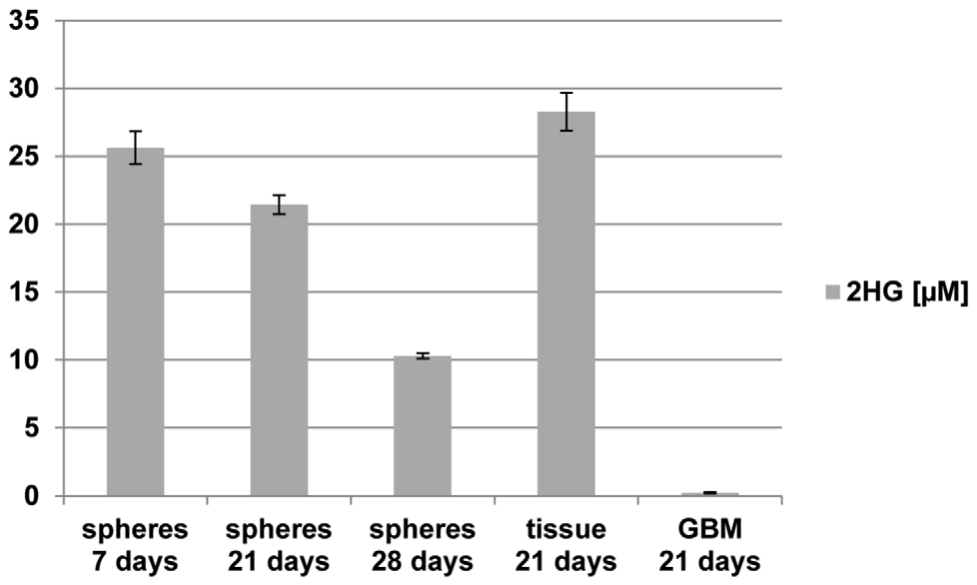
Oligodendroglioma cells produce 2HG in culture. Concentrations of 2HG in the medium from spheres and non-dissociated tissue from the xenograft relative to spheres from a freshly dissociated GBM without *IDH* mutation were measured over time. Measurements were made in triplicate. The decrease in 2HG concentrations is concomitant with the removal of approximately 50% of the spheres for implantation in between the 21- and 28-day time points.

Detection of 2HG, the metabolite synthesized by R132H, is one way to determine that the enzyme is active in the tumor cells. Using a novel enzymatic assay, we measured 2HG concentrations over time in media from re-aggregated spheres and tissue pieces from the xenograft in culture. At all time points analyzed in culture, xenograft spheres and tissue pieces exhibited measurable concentrations of 2HG while media obtained from a freshly dissociated, proliferating primary GBM cell population that did not have an *IDH1/2* mutation had no detectable 2HG (Figure 4). Levels of 2HG from the xenograft cells corresponded to those of a transfected clone that stably expresses R132H at levels equivalent to endogenous R132H protein in human tumor cells [25].

### Stability of the genetic landscape over xenograft generations

**Figure 5:**
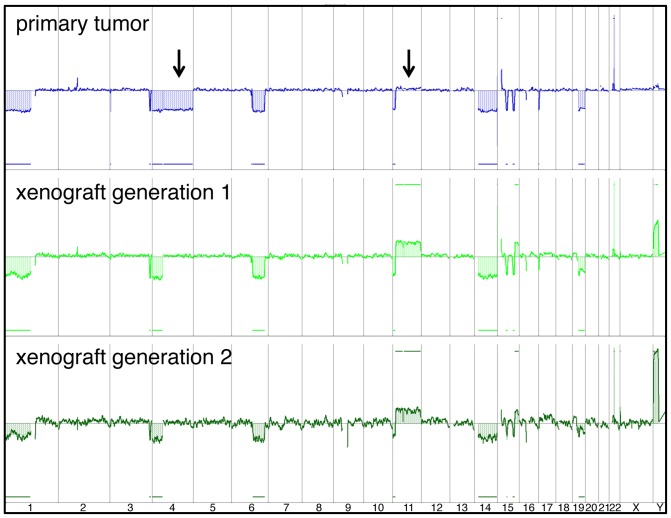
Hallmark genetic changes in the xenograft are retained over successive generations in mice. aCGH profiles for the primary human oligodendroglioma and the first and second passage xenografts are shown. Arrows highlight genetic features that differ between the primary tumor and the subsequent xenografts. Data is shown in log_2_ space across the genome where log_2_ 0 (at the midline) indicates the normal diploid copy number. Both xenograft DNAs were from male mice and the contaminating mouse DNA exhibits cross-species hybridization to the human Y chromosome.

As engraftment in animals represents a change in environment for tumor cells, selection for subpopulations or new genetic changes that deviate from the original tumor may occur. To determine whether the genetic signature of the patient tumor had been preserved in the experimental model, aCGH was performed on DNA prepared from the patient material and xenografts from the first and second passage implantations. aCGH performed on the DNA from the primary tumor showed co-deletion of 1p and 19q as well as loss of chromosomes 4, 6q16.3-q25.2, 11pter-p15.2 and 14 (Figure 5). In the xenografts, 1p and 19q losses were maintained as well as losses of chromosomes 6, 11, and 14. This pattern of loss and the exact conservation of chromosomal breakpoints on 6q or 11p confirmed that the xenograft developed from the patient sample.

Nevertheless, there were some deviations in the genetic profile of the xenograft compared to the patient tumor. Gain of most of chromosome 11 occurred. Curiously, a “gain” of chromosome 4q occurred in the xenograft so that it, once again, was diploid whereas the patient tumor was hemizygous for the entire chromosome 4.

### Preserved mutations of tumor suppressor genes in the xenograft

**Figure 6:**
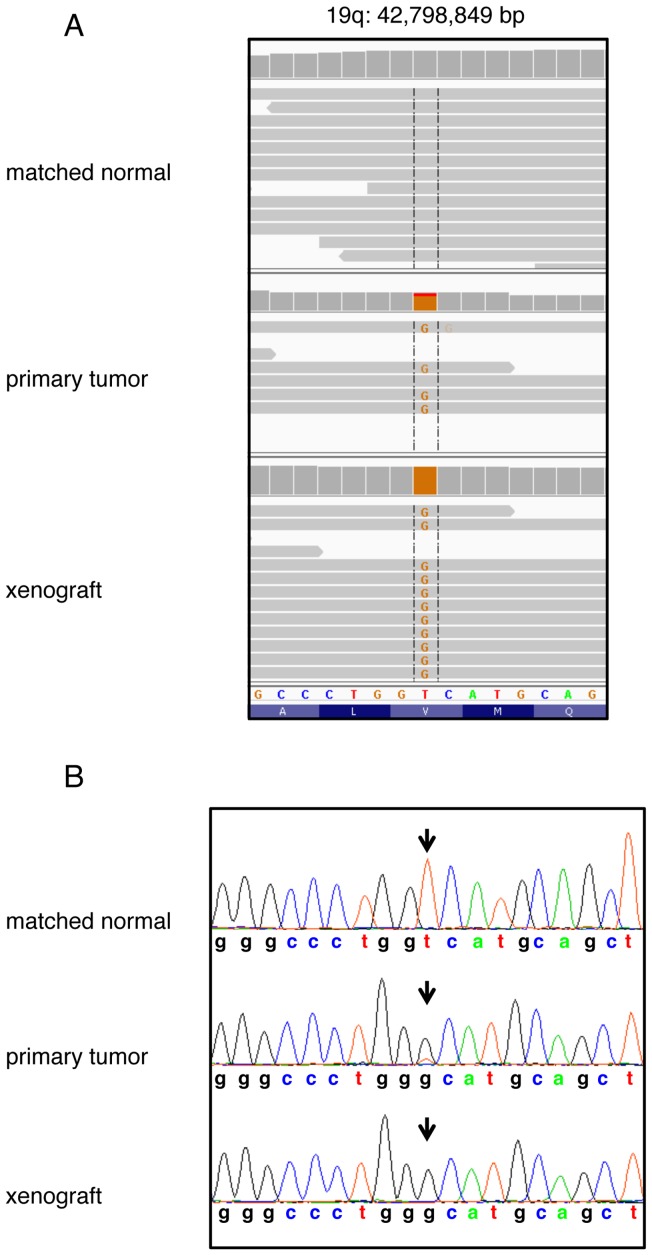
Somatic mutation of *CIC* in primary tumor and xenograft. An example of a somatic mutation of *CIC* is shown by exome sequencing of matched normal and tumor, and xenograft DNAs. (A) Coordinate genomic regions of the normal DNA from the patient and the primary tumor and xenograft are shown by exome sequencing. The increased frequency of the mutation in the reads is clearly evident in those obtained from the xenograft relative to the primary tumor. (B) The relevant base pairs are confirmed by Sanger sequencing in each of the corresponding DNAs. Arrows indicate the base pair of interest.

Exome sequencing of human oligodendrogliomas specifically with chromosome 1p and 19q losses has revealed recurrent mutations in tumor suppressor genes at these particular loci as well as generated an overall genetic map at base pair resolution [2],[3]. Typically, an oligodendroglioma with 1p and 19q losses can sustain mutations in *FUBP1* (15%, [2],[27]), *CIC* (83% [27]), *IDH1/2*, and occasionally *Notch1* or *Notch2*, whereas mutations in *p53* are absent [2], [3]. *Notch1* mutations have been shown to be activating in some cancers [28], but their role in the development of oligodendrogliomas as a tumor suppressor or oncogene is unknown. We performed exome sequencing and analyzed the obtained data to determine the status of these genes in the primary tumor and the xenograft relative to the normal patient genome. Exome sequencing confirmed the *IDH1* mutation and revealed novel mutations in *FUBP1* and *CIC* in the primary tumor as well as the xenograft relative to the normal patient genome. These mutations were subsequently confirmed by Sanger sequencing. The results for *CIC* by both sequencing methods are shown (Figure 6A, B). *FUBP1* exhibited a four base pair deletion that created a frameshift, c.1304_1307del p.Ile436Thrfs*7, and *CIC* was mutated in exon 19, c.4421T>G, p.Val1474Gly. A mutation in this codon of *CIC* has been previously reported [2]. Thus, this xenograft represents the first known to harbor mutations simultaneously in both of these newly characterized tumor suppressor genes. *p53* remained wild type even in the xenograft, and no mutations were found in *Notch1* or *Notch2* genes. Tumor suppressor genes that are more often associated with glioma progression, *CDKN2A* (9p) and *PTEN* (10q), were also not mutated in either the primary tumor or the xenograft. These results indicate that with regard to these genes, the xenograft reflects a typical oligodendroglioma with co-deletion of 1p and 19q.

### Activation of PDGFRA in the xenograft

**Figure 7:**
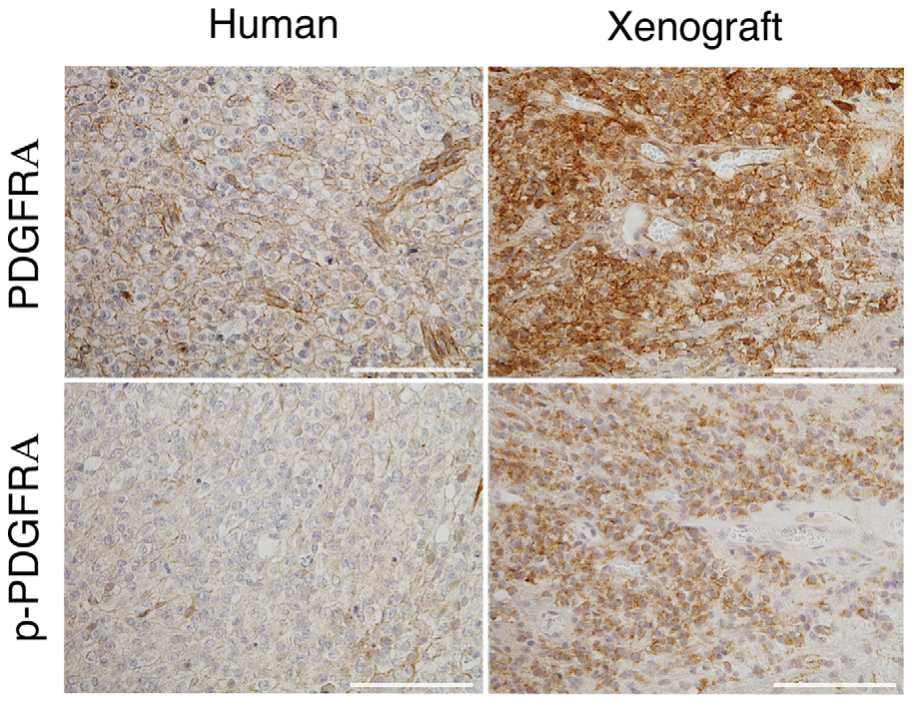
PDGFRA is activated in the xenograft. Expression of PDGFRA is detected by immunostaining of the primary human tumor and the subsequent xenograft; diffuse staining by a PDGFRA phosphorylation specific antibody in the primary human tumor, whereas intense staining is evident in the xenograft. Scale bars 100 μm.

One of the key regulators of oligodendroglioma development is the PDGFRA pathway [29],[30]. In the xenograft, as in the primary tumor, positive PDGFRA expression was seen, both in tumor cells and blood vessels (Figure 7). In addition, the receptor appeared to be activated in the xenograft based on intense positive staining with antibody against phospho-PDGFRA that was specific for the tumor cells (Figure 7). The receptor was also activated in the patient tumor, albeit to a lesser degree. Based on aCGH and exome sequencing data, the *PDGFRA* gene on 4q was neither amplified (Figure 5) nor mutated, as is known to happen in high grade oligodendroglioma [31],[32]. Thus, activation of the gene in the xenograft must be due to other mechanisms.

### Oligodendroglioma cells do not expand in culture

One of the goals of this work was to develop an *in vitro* model of human oligodendroglioma. In our xenograft, nearly 50% of oligodendroglioma cells were Ki67 positive (Figure 1F). Yet, over the course of six months, these cells, even after two passages through mice, did not expand in suspension culture in serum free medium supplemented with FGF, and EGF and/or PDGFA, as has been previously reported for other oligodendroglioma and oligoastrocytoma cell populations [21],[22].

**Figure 8:**
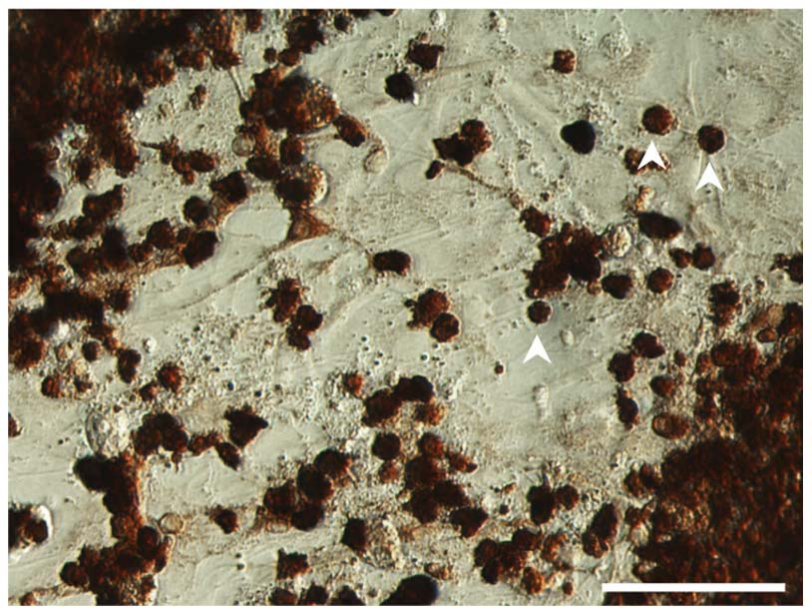
Oligodendroglioma cells attach to rat primary hippocampal neurons. Tumor cells were plated onto a bed of hippocampal neurons prepared from day old newborn rats and cultured in NBM with FGF for two weeks. Cells were fixed and incubated with an antibody specific for a human cytoplasmic protein, STEM121. Arrows point to individual cells. Scale bar 50 μm.

The behavior of the oligodendroglioma cells was also examined under adherent conditions. The tumor cells never attached to fibronectin or laminin coated slides. Because this tumor cell population accumulated around neurons in the xenografts, we investigated whether the spheres would attach to this cell type *in vitro*. The oligodendroglioma spheres were highly adherent to newborn rat hippocampal neurons, as opposed to fibronectin or laminin alone. Individual cells migrated away from spheres attached to the neurons; however, cell expansion was not evident over the time period of two weeks (Figure 8).

**Figure 9:**
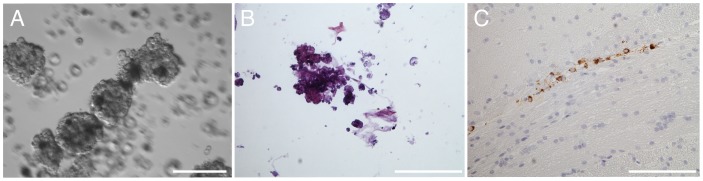
Anaplastic oligodendroglioma that did not develop tumors *in vivo* and shows calcifications *in vitro*. (A) Spheres that formed from dissociated material of a second anaplastic oligodendroglioma; (B) calcified sphere after > three months in culture; (C) tumor cells evident in a single animal at 103 days through immunostaining with STEM121. Scale bars A 50 μm, B and C 100 μm.

We were also unable to expand a second primary recurrent anaplastic oligodendroglioma, with 1p/19q co-deletion, over an extended period of time in suspension culture (Figure 9A). This tumor cell population, in fact, exhibited an extreme growth arrest phenotype as we observed spheres to progressively turn into calcium crystals *in vitro* (Figure 9B). Spheres did not generate xenografts, although some cells could be detected in the corpus callosum of a single animal (one out of nine animals; Figure 9C). We also observed some calcified cells from our model when placed in culture, but to a lesser degree. This result parallels an interesting common histological feature of oligodendrogliomas, calcifications, the origin of which is currently unknown. Sphere cultures were prepared similarly from four grade II oligodendrogliomas with 1p/19q co-deletion. None expanded in culture, and engraftment was not evident with two that were implanted (data not shown). In contrast to oligodendrogliomas, we readily established proliferating sphere cultures from eight out of nine primary GBM specimens under identical conditions *in vitro* (data not shown).

### Characterization of the stroma

**Figure 10:**
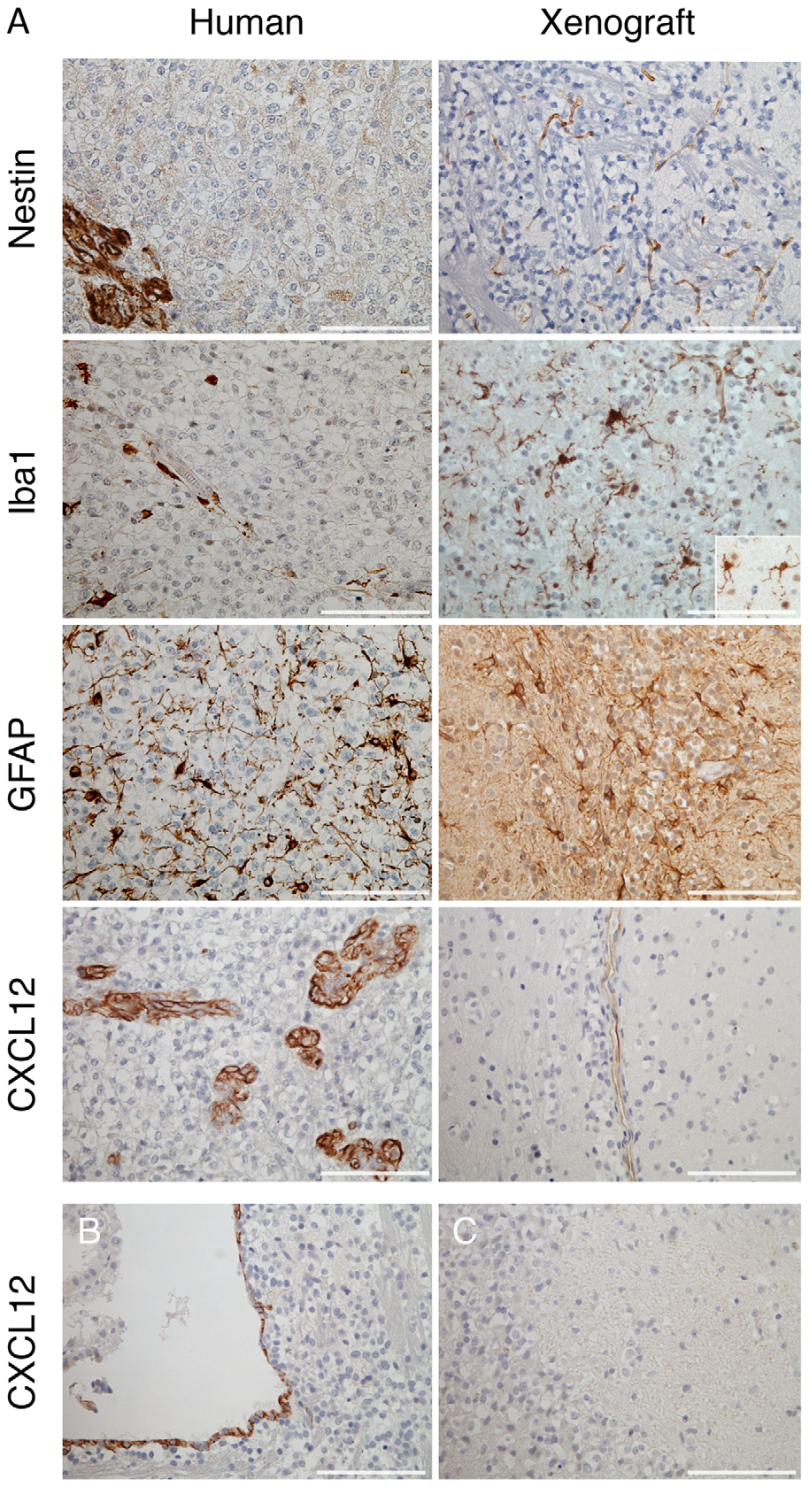
Astrocytes intermingled in the tumor bed of both the primary tumor and the xenograft. (A) Sections of the primary human tumor and xenograft were immunostained for nestin, Iba1, and GFAP to observe infiltration by neural stem or progenitor cells, microglia/macrophages, and astrocytes, respectively. Inset in Iba1 staining displays morphology of microglia on the contra-lateral side of the mouse xenograft at the same magnification. CXCL12 staining was performed on sections from the xenograft shown in Figure 3B and E. Vessels from the primary tumor and xenograft are shown as well as (B) the subventricular zone and (C) the hippocampus of the xenograft. Scale bars 100 μm.

To begin to understand the nature of the apparent synergy between the *in vivo* host environment and this tumor cell population, we looked for evidence of relationships that might exist between the tumor cells and specific stromal cell types. Perhaps the most unique feature of this particular model is the assembly of tumor cells around neurons (Figure 3B, C). However, large areas of the original patient tumor were expanding independently of neurons, and furthermore, co-culture of the tumor cells with neurons *in vitro* did not result in enhanced growth (Figure 8). Some human glioma cells in xenografts migrate into the ventricles where stem cell populations reside or attract these cells to themselves [33]. To identify normal stem cells present within the primary tumor or xenograft, we performed immunohistochemistry with antibodies specific for human or mouse nestin. In the xenograft, we observed nestin positive mouse neural stem cells in the ventricles, where expected, but no positively stained cells were found within the tumor bed except for the vasculature. This observation is consistent with previous findings [34],[35],[36],[37]. Intense staining of the vasculature was also seen in the human tumor, but no other nestin positive cells, not even the tumor cells themselves, were observed within the tumor bed (Figure 10A). Immune cell types such as microglia/macrophages have been shown to promote the growth of human gliomas [38]. In the patient tumor, microglia/macrophages were most concentrated at the periphery of areas of highly proliferating tumor cells (Figure 10A). Microglia did not accumulate or increase in number within the xenograft (Figure 10A), but some exhibited a hypertrophic morphology as opposed to those in the contra-lateral hemisphere (Figure 10A inset). Finally, we stained specimens for the astrocyte marker GFAP as astrocytes are found throughout the brain around neurons and in the CNS vasculature. Astrocytes appeared to be highly embedded within the patient tumor as well as in the xenograft (Figure 10A).

Because the tumor cells in the xenografts migrated to specific regions of the brain or along anatomical structures, including perivascular, perineuronal, and subependymal regions, we examined these areas for the expression of the chemokine CXCL12. Normal neural stem cells have been shown to home to and migrate from the ependymal layer in the subventricular zone (SVZ) and vascular niches in response to CXCL12 [39]. CXCL12 was highly expressed in the vessels of the primary tumor and to a more limited degree, in the larger vessels of the xenograft (Figure 10A). CXCL12 was also detected in ependymal cells lining the ventricle walls of a xenograft but not in the hippocampus infiltrated by tumor cells (Figure 10B, C). These results indicate that CXCL12 may potentially drive the migration of these tumor cells to the larger vessels or the ependymal layer where they may more efficiently proliferate.

## Discussion

We have successfully established and serially passaged a grade III oligodendroglioma xenograft with the hallmark chromosome 1p and 19q losses and mutations including *FUBP1*, *CIC*, and *IDH1* (R132H). Although this tumor had a high growth fraction, as measured by Ki67 staining, we were unable to expand the original patient tumor *in vitro*. Furthermore, none of the gross genetic changes in the xenograft that differ from the original tumor, such as gain of chromosomes 4q or 11, enhanced *in vitro* growth. These results together support investigation of the brain environment for components and/or conditions that are necessary for the continued maintenance of this human oligodendroglioma.

Several crucial questions are raised by this work. The first is what is the genetic basis for the engraftment. Based on aCGH data and limited analysis of exome sequencing, the tumor reported here has a common anaplastic oligodendroglioma genomic profile including *FUBP1*, *CIC*, and *IDH1* mutations. Equally informative is that there is an absence of mutations in *p53*, *PTEN*, or *CDKN2A* that can accompany glioma progression [31]. With regard to these mutations, this result is especially surprising as this case was a recurrent, treated oligodendroglioma. It could be expected that a subpopulation with mutation in *PTEN* or *CDKN2A* might have been selected for, particularly in the xenograft. In the absence of these mutations and a complete analysis of the exome sequencing, one possibility is that it is the combination of *CIC* and *FUBP1* mutations that provide this tumor with an advantage during engraftment. In fact, while mutation of *CIC* is a more common denominator amongst oligodendrogliomas, mutation of *FUBP1* is not. The percentage of oligodendrogliomas with *CIC* mutations is in the range of 50 to 80% [2],[3],[27],[40], whereas *FUBP1* mutations have been found to occur in approximately 15% to 20% of oligodendrogliomas in three studies [2],[27],[40]. Twenty-one mutations including this one have been reported in 103 oligodendrogliomas with fourteen of the *FUBP1* mutations in anaplastic cases [2],[27],[40]. Only twelve of these tumors actually have concomitant mutations in *FUBP1* and *CIC*, and nine of these cases are anaplastic oligodendrogliomas. *FUBP1* regulates *MYC* expression, a gene that is known to induce proliferation and pluripotency in different cell types [41]. It is possible that this combination of *FUBP1* and *CIC* mutations, in the absence of other known major gene mutations, gave this particular tumor some kind of growth advantage upon intracranial implantation. However, analysis of additional engrafted cases and deeper analysis of the sequencing dataset is necessary to more fully understand the success of intracranial engraftment with regard to oligodendrogliomas.

In addition to the underlying genome, our timeframe of nearly one year was considerably longer than other studies that terminate *in vivo* experiments at sixteen weeks or even less [21],[42]. An additional study has reported intracranial engraftment after five to eight months, but the xenografts were not characterized genetically nor was histology presented [43]. Although Ki67 demonstrated that a high percentage of cells were in the growth fraction of the xenografts we report here, this result is not a measure of the cell cycle time of individual cells. The fact that mutations in *p53*, *CDKN2A*, and *PTEN* more rarely occur in oligodendrogliomas might indicate a slower growing cell type [44]. If so, the animals must live long enough to exhibit tumor pathology.

A second important question is what promotes proliferation and/or survival of the xenograft *in vivo*. That these tumor cells preferentially grow *in vivo* corroborates results recently obtained with an anaplastic oligoastrocytoma with a similar genetic constellation of 1p and 19q losses and R132H [22]. A simple scenario for a cooperative effect between these tumor cells and the *in vivo* environment would be that a specific growth factor/receptor enhances proliferation and/or survival. The *PDGFRA* gene presents one potential *in vivo* candidate for the xenograft, but the status of this gene exhibits a peculiar behavior. The primary tumor actually has loss of chromosome 4q where the *PDGFRA* gene is located, whereas the xenograft becomes diploid again at this chromosomal arm. This loss is frequently observed in oligodendrogliomas, generally in higher grade tumors [31]. No other recurrent mutations have been identified in genes on chromosome 4q in oligodendrogliomas [2],[3].

A final question is from where such a stimulatory signal may originate. Here, we initiated investigation of cell types from the stroma as potential candidates. The results of our experiments for immunostaining for the presence of specific cell types within the xenografts showed surprisingly few differences from normal brain, other than subtle changes in microglia morphology and accumulation of astrocytes. Additional clues for what supports the growth of this oligodendroglioma may, instead, be obtained by studying the areas within the CNS where the tumor xenografts grew. The most striking and unique pattern of tumor cell growth was around hippocampal neurons. Infiltration along neurons is a common histological feature of human oligodendroglioma, but preliminary results from our laboratory indicate that *in vitro*, neuronal cultures do not support expansion of oligodendroglioma cells. The second pattern of *in vivo* growth observed was around established vasculature. Tumor cell growth, as assessed by Ki67 immunostaining, was strong around blood vessels in the cortex, the striatum, and particularly within the subgranular zone (SGZ) of the dentate gyrus in one of the xenografts. The result of growth, specifically in the SGZ, is particularly interesting as it is an area where normal neural progenitor cells are known to form highly proliferative clusters in conjunction with endothelial cells [45]. From these studies, the concept of the vascular niche as crucial to the maintenance and proliferation of normal neural stem and progenitor cells has emerged. Thus, the fact that oligodendroglioma tumor cells accumulate and/or thrive in reported vascular niches recapitulates some aspects of normal stem and progenitor cell behavior, and efforts to define components within these niches may help us to understand the development of human oligodendroglioma [46].

In conclusion, our results show for the first time that histological and genetic features, such as satellitosis and expression of R132H, of a human oligodendroglioma are maintained in an intracranial mouse xenograft over serial passages. Future efforts to develop critically needed *in vivo* oligodendroglioma models, that more broadly represent the disease, will help us to define the environmental parameters contributing to oligodendroglioma development and growth and may open up new avenues of therapeutic value.
