# Correction: A Novel, Diffusely Infiltrative Xenograft Model of Human Anaplastic Oligodendroglioma with Mutations in *FUBP1*, *CIC*, and *IDH1*

**DOI:** 10.1371/annotation/72a01ce5-b0ad-4cbd-ac77-0a81347a3940

**Published:** 2013-08-06

**Authors:** Barbara Klink, Hrvoje Miletic, Daniel Stieber, Peter C. Huszthy, Jaime Alberto Campos Valenzuela, Jörg Balss, Jian Wang, Manja Schubert, Per Øystein Sakariassen, Terje Sundstrøm, Anja Torsvik, Mads Aarhus, Rupavathana Mahesparan, Andreas von Deimling, Lars Kaderali, Simone P. Niclou, Evelin Schröck, Rolf Bjerkvig, Janice M. Nigro

The name of the fifth author was given incorrectly in the citation. The correct citation is: Klink B, Miletic H, Stieber D, Huszthy PC, Campos Valenzuela JA, et al. (2013) A Novel, Diffusely Infiltrative Xenograft Model of Human Anaplastic Oligodendroglioma with Mutations in *FUBP1*, *CIC*, and *IDH1*. PLoS ONE 8(3): e59773. doi:10.1371/journal.pone.0059773. 

